# Links Between Plant Functional Reorganization and a Soil Multifunctionality Proxy During Karst Forest Recovery

**DOI:** 10.3390/plants15162448

**Published:** 2026-08-12

**Authors:** Yang Wang, Yangyang Ji, Juan Tao, Wanchang Zhang, Jintong Ren, Hongju Wang, Ruiyu Zhou, Xiu Huang, Xueyi Fang, Dengchuan Li

**Affiliations:** 1 School of Ecological Engineering, Guizhou University of Engineering Science, Bijie 551700, China; 15125497829@163.com (Y.J.); 18484643869@163.com (J.T.); zhangwc@radi.ac.cn (W.Z.); renjintong@gues.edu.cn (J.R.); 2131931056@163.com (H.W.); 18085091257@163.com (R.Z.); 19886894351@163.com (X.H.); 17385681438@163.com (X.F.); wqgzldc@163.com (D.L.); 2 Guizhou Key Laboratory of Plateau Wetland Conservation and Restoration, Bijie 551700, China

**Keywords:** karst landscape, leaf economic spectrum, functional diversity, soil multifunctionality proxy, functional reorganization

## Abstract

Whether vegetation structural recovery is accompanied by improvement in soil properties remains a key question for evaluating karst forest restoration. We investigated a six-stage natural recovery sequence in the Maolan karst forests of southwestern China by analyzing 18 plots. This sequence was interpreted as a spatial gradient rather than a strict temporal succession. Six soil physicochemical variables, community-weighted leaf traits, and functional diversity indices were integrated to construct a relative soil multifunctionality proxy (SMF) and to evaluate associations between soil-property patterns and plant functional reorganization. Later woody stages generally had lower soil bulk density and higher soil moisture, organic carbon, nitrogen, phosphorus, and potassium contents, and the SMF was higher from the tree–shrub stage onward. Community-weighted leaf traits showed nonlinear reorganization along the leaf economic spectrum, indicating shifts in resource acquisition and tissue investment strategies. Functional diversity responded asynchronously: functional richness was positively associated with the SMF, whereas dispersion-related indices did not show consistent positive associations. These findings indicate that variation in the SMF is associated with measured soil properties, the reorganization of dominant leaf traits along the leaf economic spectrum, and the restructuring of functional trait space, rather than being attributable to any single trait or diversity index. Because this study used 18 plots and a space-for-time design, the observed relationships should be interpreted as spatial associations, not temporal or causal effects.

## 1. Introduction

Ecological restoration research is progressively shifting from a sole focus on vegetation cover toward the recovery of ecosystem functions. In degraded forests, successful restoration depends not only on re-establishing species composition and community structure but also on recovering functions such as carbon sequestration, nutrient cycling, water retention, and soil stability [1,2]. Multifunctionality provides a framework for assessing multiple ecological processes simultaneously, but the indicators selected must correspond to the study system and the variables measured [3,4]. When measurements primarily describe soil physical structure, moisture status, and carbon and nutrient contents, a soil multifunctionality proxy (SMF) provides a more specific operational summary than broader ecosystem multifunctionality [5]. Recent forest research further indicates that tree spatial arrangement, canopy structure, and community age can influence the maintenance of multifunctionality [6,7]. Clarifying how the SMF differs among forest recovery stages is therefore important for evaluating whether vegetation restoration corresponds to improved soil-property conditions.

Karst regions in southwestern China are characterized by shallow soils, extensive bedrock exposure, and slow soil formation. These ecosystems are highly sensitive to land-use disturbance and soil erosion [8]. Although ecological engineering and natural recovery have enhanced vegetation growth and carbon sequestration in karst landscapes, vegetation greening does not necessarily correspond to concurrent improvements in soil structure, moisture status, and nutrient availability [9]. Patchy soils, rapid fissure drainage, and limited nutrient resources can impede early plant establishment and produce stage-dependent, nonlinear trajectories in soil-property recovery [2,10]. The success of karst restoration is also influenced by lithology, bedrock exposure, and soil erosion dynamics [11,12]. Changes in soil physicochemical properties and microbial community composition are increasingly recognized as important foundations for functional recovery during vegetation restoration in karst environments [13,14]. Assessments based only on vegetation cover or species richness are therefore incomplete; a more comprehensive evaluation should integrate soil resource status, plant functional traits, and a clearly delimited soil multifunctionality proxy. Unlike conventional soil quality indices that commonly integrate physical, chemical, and biological indicators through weighted scoring systems, our soil multifunctionality (SMF) proxy is explicitly confined to six measured soil physicochemical variables and utilizes a transparent, unweighted averaging method. Furthermore, it is intentionally more limited in scope than ecosystem multifunctionality indices, which generally incorporate multiple ecosystem processes across trophic levels using averaging or threshold-based approaches. This focused definition prevents overstating the functional coverage beyond what our data support and ensures straightforward interpretability of soil property patterns along the recovery gradient.

Plant functional traits link environmental filtering, community assembly, and ecosystem functioning [15,16]. Leaf traits, in particular, characterize trade-offs in resource acquisition, tissue construction, and nutrient use and can therefore reveal shifts in dominant plant strategies during ecological recovery. The leaf economic spectrum describes a continuum from rapid resource acquisition to conservative resource use and is commonly represented by specific leaf area, leaf dry matter content, leaf nitrogen content, and leaf phosphorus content [17,18]. Across restoration sequences, changes in community-weighted leaf traits reflect species turnover and environmental filtering. These traits may also be related to soil properties through litter quality, rhizosphere inputs, and nutrient cycling [19]. Recent research indicates that dominant-species photosynthetic traits, leaf-trait variability, and leaf-associated microbial communities can modulate community functional performance [20,21]. In karst plantations, often-overlooked petiole traits may also be associated with soil carbon sequestration [22]. Nevertheless, evidence remains limited on whether reorganization of the leaf economic spectrum accompanies changes in an integrated soil multifunctionality proxy across a complete karst forest recovery sequence.

Functional diversity encompasses variation in traits, niche differentiation, and functional redundancy within ecological communities, extending beyond dominant trait values. It is therefore an important dimension for interpreting how plant communities relate to ecosystem functioning. Functional richness, functional evenness, functional divergence, functional dispersion, and Rao’s quadratic entropy characterize, respectively, the volume of functional trait space, the evenness of trait distribution, divergence toward the periphery of trait space, average distance from the centroid, and abundance-weighted trait dissimilarity [23,24]. During recovery, expansion of functional space may enhance resource-use complementarity, whereas environmental filtering in later stages and the establishment of dominant woody species may produce convergence in some traits. Assembly history, habitat filtering, and dispersal limitation can alter functional diversity and its ecological consequences during forest recovery [25,26]. We therefore investigated a six-stage, space-for-time natural recovery sequence in the Maolan karst forests. We constructed a relative, sample-dependent SMF from soil physical structure, moisture status, and carbon and nutrient contents and integrated it with community-weighted leaf traits, functional diversity indices, and multivariate analyses. We asked: (1) whether later recovery stages were associated with higher SMF values through lower soil bulk density and higher soil moisture and carbon and nutrient contents, and whether a pronounced stage-related shift occurred; and (2) whether reorganization of the leaf economic spectrum and restructuring of functional diversity were differentially associated with the SMF and individual soil properties. This approach was intended to clarify soil–plant functional associations and develop testable mechanistic hypotheses for function-oriented management of rocky desertification.

## 2. Results

### 2.1. Soil-Property Patterns and the SMF Along the Recovery Sequence

**Figure 1:**
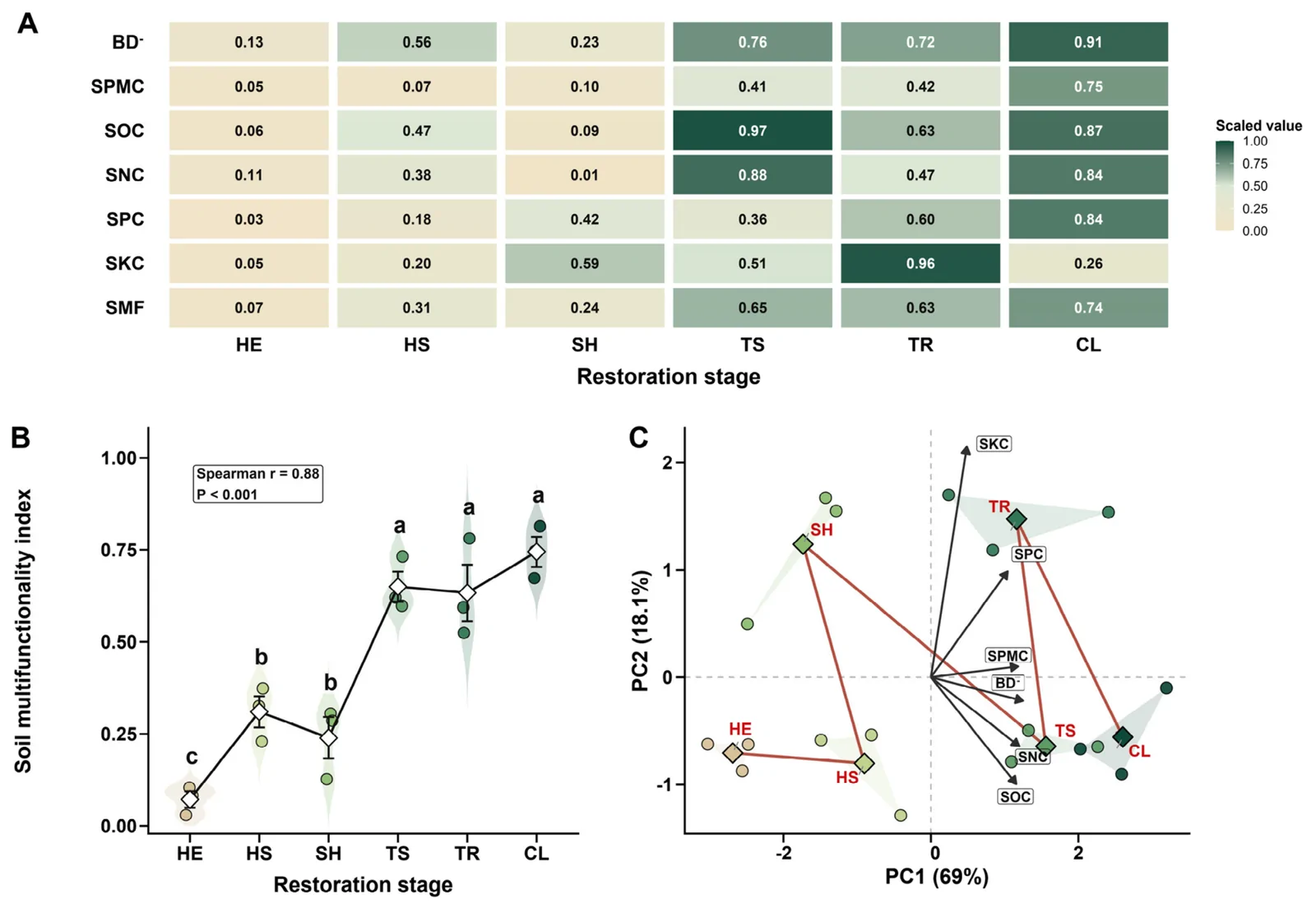
Soil property patterns and the soil multifunctionality (SMF) proxy during karst forest recovery are presented as follows: (**A**) A heatmap illustrating Z-standardized mean values across stages for six soil indicators, with BD^−^ representing reverse-normalized bulk density. (**B**) A boxplot depicting SMF values across different stages; individual points correspond to plot-level values, while diamonds and error bars represent means ± standard error. Distinct letters denote statistically significant differences (*p* < 0.05), determined by Tukey’s HSD or rank-based pairwise comparisons with false discovery rate adjustment. The effect size for the stage comparison was large (η^2^ = 0.954), indicating substantial biological significance of the observed differences. (**C**) Principal component analysis (PCA) ordination of six soil variables (PC1 = 69.0%, PC2 = 18.1%); brown-red lines connect stage centroids, and gray arrows show variable loadings. In addition, the SMF calculated using PCA-derived weights was nearly perfectly correlated with the equal-weight SMF (Spearman’s *ρ* = 0.99, *p* = 9.93 × 10^−6^), confirming that the main findings were robust to the choice of weighting approach.

Soil properties showed a distinct stage-related pattern across the karst forest recovery sequence (Figure 1). The Z-standardized heatmap indicated low soil moisture and nutrient contents in the early stages, with mean SMF values of 0.07 in grassland (HE) and 0.27 in HS. Most indicators were higher from the tree–shrub stage onward, with mean SMF values of 0.60 in tree–shrub (TS), 0.61 in tree (TR), and 0.74 in near-climax community (CL) (Figure 1A). The actual (non-standardized) values of all soil properties across stages are provided in Supplementary Table S1. Soil bulk density showed the opposite pattern, as indicated by higher reverse-normalized bulk density (BD^−^) in later stages. Soil organic carbon (SOC) and soil nitrogen content (SNC) were relatively high in TS and CL, whereas soil potassium content (SKC) peaked in TR. The SMF showed a generally increasing stage-related pattern and was strongly correlated with the ordered recovery stage (Spearman r = 0.88, *p* < 0.001; Figure 1B). In the soil-property PCA, PC1 and PC2 explained 69.0% and 18.1% of the variance, respectively. The centroid trajectory shifted from HE and grass–shrub (HS) toward TS, TR, and CL, indicating a transition from early resource-poor plots to later plots with higher integrated soil-property scores (Figure 1C).

### 2.2. Leaf Economic Spectrum Reorganization and Dominant Trait Changes

**Figure 2:**
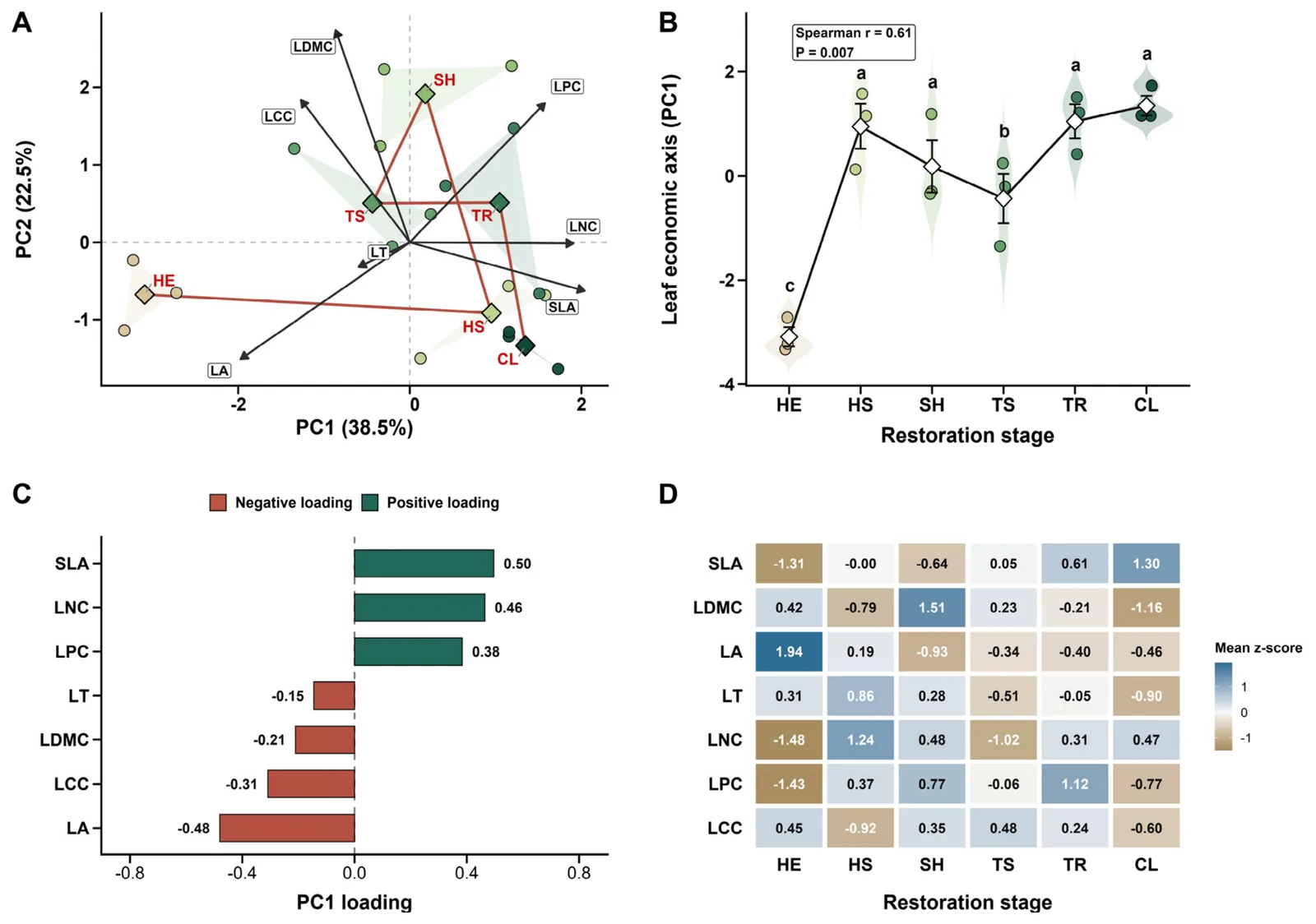
Reorganization of community-weighted leaf traits throughout the recovery sequence is presented. (**A**) Principal Component Analysis (PCA) ordination of community-weighted leaf traits (PC1 = 38.5%, PC2 = 22.5%), with stage centroids and trajectories. (**B**) Variation in leaf economic PC1 across stages; points and diamonds correspond to those in Figure 1B. Different letters denote statistically significant differences (*p* < 0.05). The effect size for the stage comparison was large (η^2^ = 0.890), indicating substantial biological significance of the observed differences. (**C**) Trait loadings on PC1. (**D**) Stage means of Z-standardized community-weighted traits. Abbreviations: SLA, specific leaf area; LDMC, leaf dry matter content; LA, leaf area; LT, leaf thickness; LCC, LNC, LPC, leaf carbon, nitrogen, and phosphorus contents.

Community-weighted leaf traits were reorganized across the recovery sequence (Figure 2). Principal component analysis (PCA) of these traits explained 61.0% of the total variation, with PC1 and PC2 accounting for 38.5% and 22.5%, respectively, and separated early-stage HE plots from later stages in leaf-trait space (Figure 2A). Leaf-trait PC1, used as an integrated leaf economic axis, increased with recovery stage (Spearman r = 0.61, *p* = 0.007), although the centroid trajectory was nonlinear rather than a simple directional progression (Figure 2B). Specific leaf area (SLA), leaf nitrogen content (LNC), and leaf phosphorus content (LPC) loaded positively on PC1 (0.50, 0.46, and 0.38, respectively), whereas leaf area (LA), leaf carbon content (LCC), leaf dry matter content (LDMC), and leaf thickness (LT) loaded negatively; LA had the strongest negative loading (−0.48; Figure 2C). The heatmap further showed asynchronous trait variation: SLA increased from HE (−1.31) to CL (1.30), LDMC peaked in shrub (SH) (1.51) and declined in CL (−1.16), LNC was highest in HS (1.24), and LPC peaked in TR (1.12). Recovery was therefore associated with stage-specific reorganization of dominant trait combinations rather than uniform change in every leaf trait (Figure 2D).

### 2.3. Multidimensional Functional Diversity and Functional-Space Reorganization

**Figure 3:**
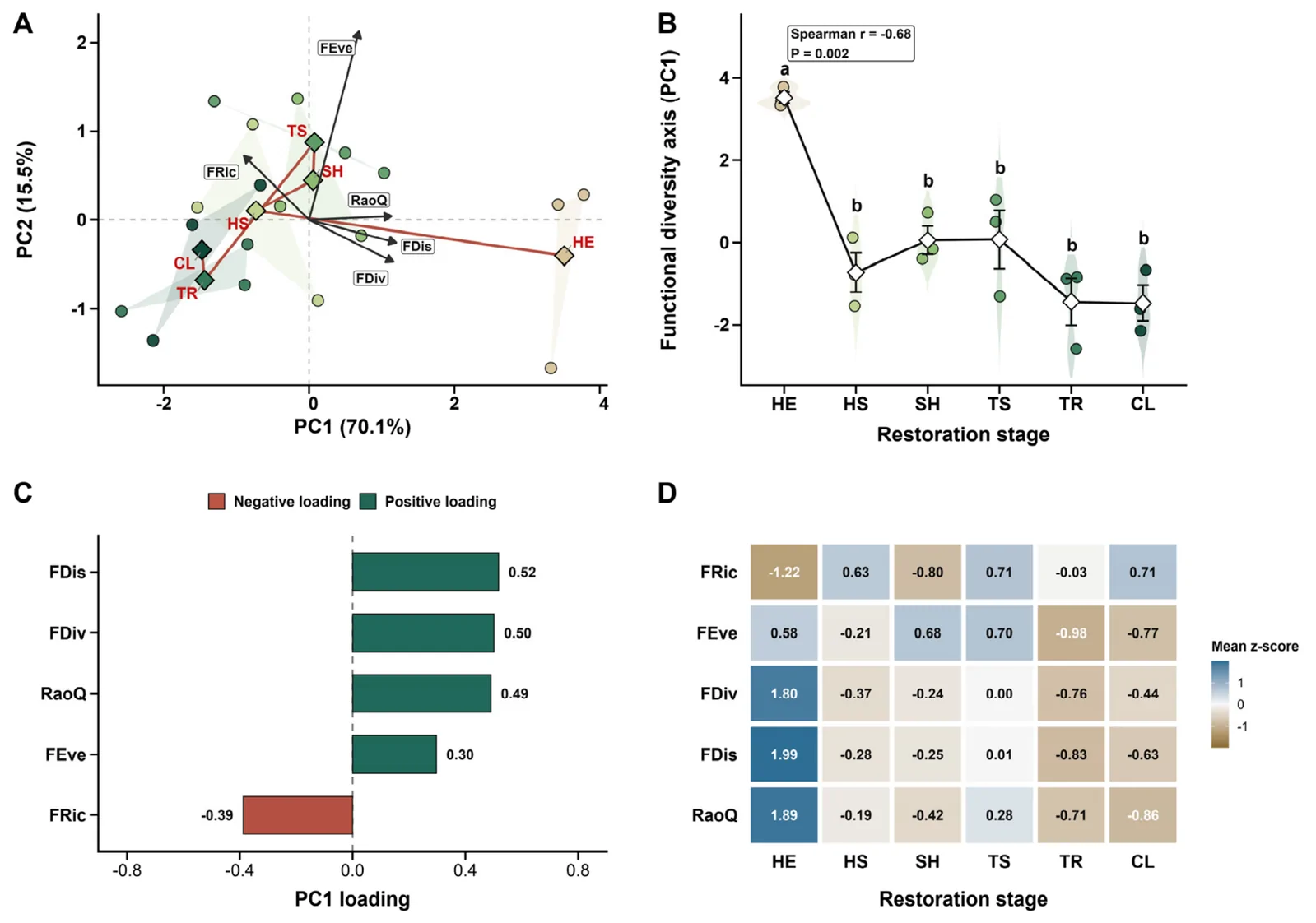
Functional diversity responses during the recovery of karst forests are presented as follows: (**A**) Principal Component Analysis (PCA) ordination of functional-diversity indices (PC1 = 70.1%, PC2 = 15.5%), with stage centroids and trajectories; (**B**) Functional diversity PC1 values across different recovery stages, with points and diamonds corresponding to those in Figure 1B. Different letters denote statistically significant differences (*p* < 0.05). The effect size for the stage comparison was large (η^2^ = 0.861), indicating substantial biological significance of the observed differences; (**C**) Loadings of indices on PC1; and (**D**) Stage means of Z-standardized indices. Abbreviations used include FRic (functional richness), FEve (functional evenness), FDiv (functional divergence), FDis (functional dispersion), and RaoQ (Rao’s quadratic entropy).

Functional diversity showed clear stage-related reorganization across the recovery sequence (Figure 3). PCA of functional richness (FRic), functional evenness (FEve), functional divergence (FDiv), functional dispersion (FDis), and RaoQ explained 85.6% of the total variation, with PC1 and PC2 accounting for 70.1% and 15.5%, respectively. Functional-diversity PC1 separated HE from later stages and was positively associated with FDis, FDiv, and RaoQ but negatively associated with FRic (Figure 3A,C). Higher original PC1 scores therefore represented greater trait dispersion, divergence, and abundance-weighted dissimilarity, whereas lower scores represented reduced dispersion and relatively greater functional-space volume.

The original functional-diversity PC1 declined along the ordered recovery sequence (Spearman r = −0.68, *p* = 0.002), with HE having the highest scores and later stages forming a more compressed cluster (Figure 3B). The heatmap showed that FRic increased in several later stages, whereas FDis, FDiv, and RaoQ generally declined after HE (Figure 3D). Karst forest recovery was therefore characterized by the expansion of functional space in some dimensions together with reduced trait dispersion and dissimilarity in others.

### 2.4. Associations of Leaf Traits and Functional Diversity with the SMF

**Figure 4:**
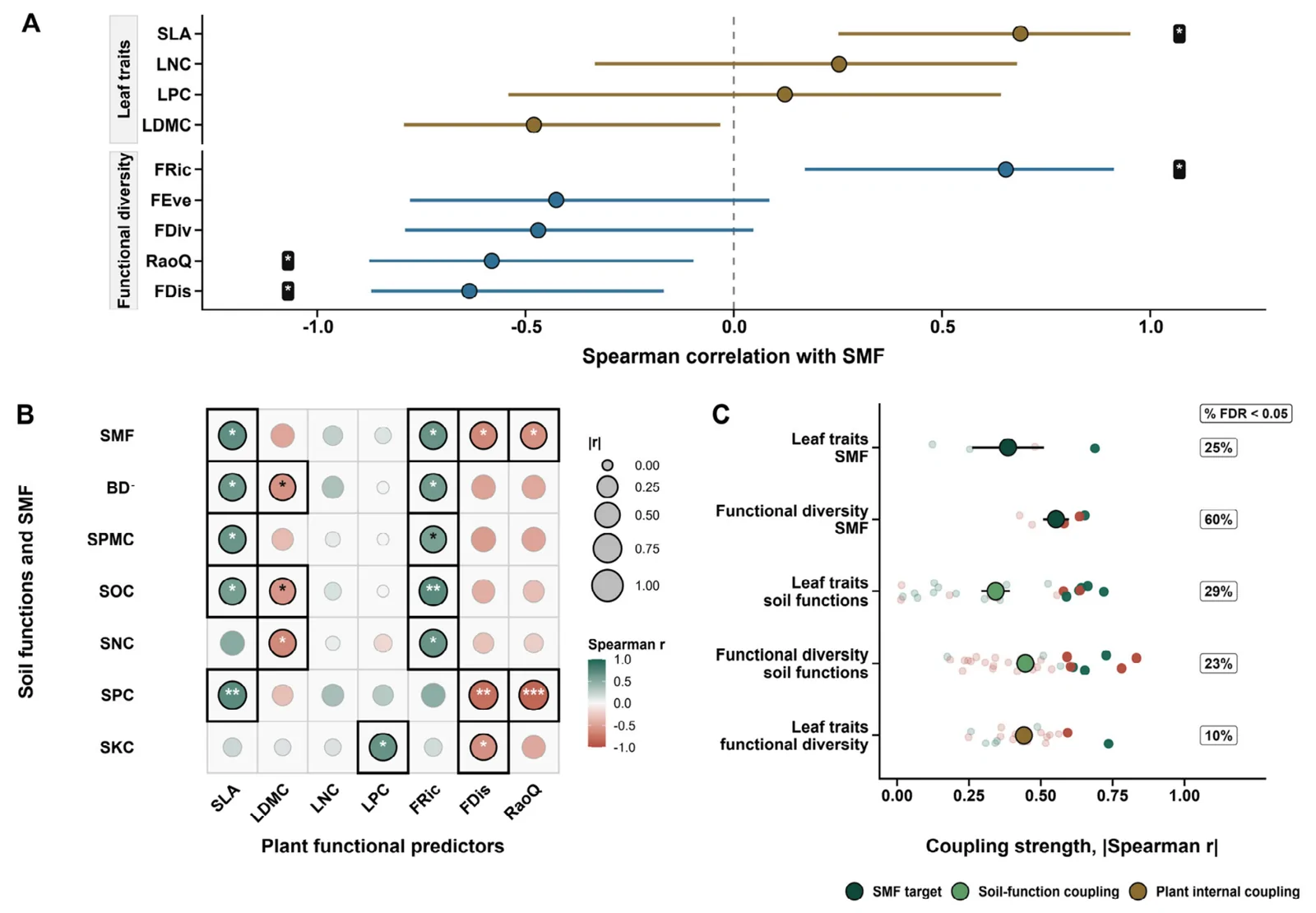
Spearman correlation analyses were conducted to examine the relationships between plant functional indicators and soil properties. (**A**) A forest plot illustrating correlations with soil multifunctionality proxy (SMF); points represent correlation coefficients, and horizontal lines denote 95% confidence intervals. (**B**) A correlation matrix depicting associations among leaf traits (**left**), functional diversity indices (**right**), and individual soil properties; the color and size of the bubbles correspond to the direction and magnitude of the correlations, respectively. Statistical significance was determined using the Benjamini–Hochberg false discovery rate (FDR) correction. * *p* < 0.05, ** *p* < 0.01, *** *p* < 0.001. (**C**) A module-level summary of cross-module correlations, where bar heights represent the mean absolute correlation coefficients, and the numbers above the bars indicate the proportion of comparisons that were significant after FDR adjustment.

Plant functional indicators showed indicator-specific associations with the SMF and individual soil properties (Figure 4). In the correlation forest plot, SLA and FRic were the only plant functional indicators with strong, positive, statistically significant associations with the SMF. FDis and RaoQ were significantly negatively correlated with the proxy, whereas LNC, LPC, LDMC, FEve, and FDiv had weaker or non-significant associations (Figure 4A). The correlation matrix further showed that plant indicators were not uniformly associated with all soil properties. SLA was positively correlated with SMF, reverse-normalized bulk density (BD^−^), soil gravimetric moisture content (SPMC), SOC, and SPC, whereas FRic was positively associated with SMF, BD^−^, SPMC, SOC, and SNC. LDMC was negatively correlated with several soil-property indicators, including BD^−^, SOC, and SNC, and RaoQ had a strong negative association with SPC (Figure 4B). Module-level analysis supported this selective pattern: functional diversity had the highest proportion of significant associations with SMF (60%), whereas leaf traits had a lower proportion (25%). Significant associations with individual soil properties accounted for 29% of leaf-trait comparisons and 23% of functional-diversity comparisons; only 10% of leaf-trait–functional-diversity comparisons were significant (Figure 4C).

### 2.5. Integrated Associations Among Functional Modules and the SMF

**Figure 5:**
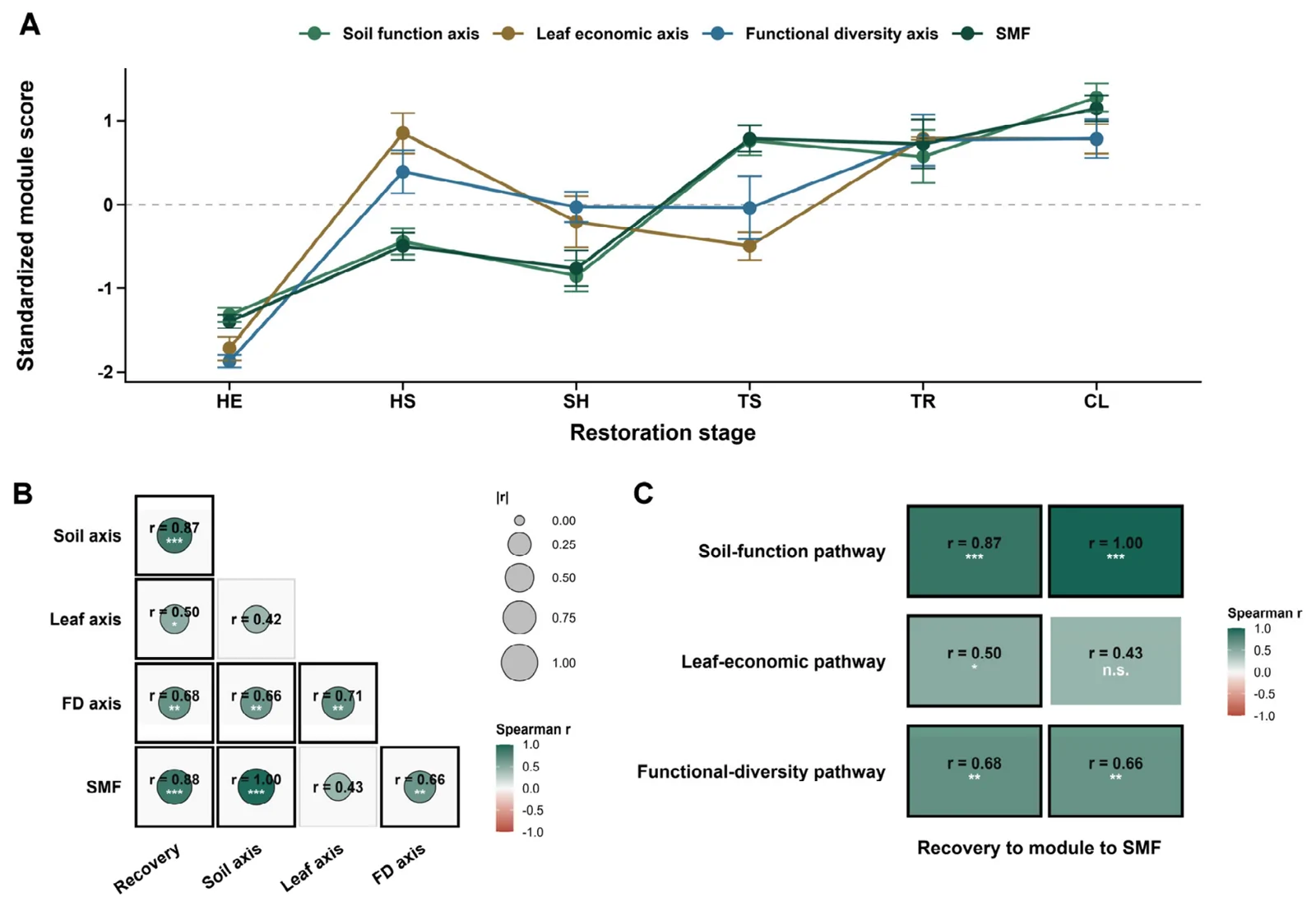
An integrated synthesis of the recovery stage, soil properties, plant functional modules, and soil multifunctionality (SMF) is presented. (**A**) Stage-specific scores are shown for the soil-property principal component analysis (PCA) axis, leaf economic spectrum principal component 1 (PC1), display-oriented functional diversity PC1, and SMF. (**B**) A Spearman correlation matrix illustrates the relationships among all modules and recovery stages. (**C**) A conceptual summary of these associations is provided. Note that the functional diversity PC1 was multiplied by −1 solely for visualization purposes; this transformation does not affect the correlation magnitudes or *p*-values. * *p* < 0.05, ** *p* < 0.01, *** *p* < 0.001, n.s. not significant. It is important to emphasize that the observed relationships represent statistical associations and do not imply causality. The perfect correlation (r = 1.00) between the soil-property axis and SMF arises from their mathematical non-independence, as both metrics are derived from the same six variables.

The integrated synthesis showed coordinated but asynchronous changes among recovery stage, soil properties, plant functional structure, and the SMF (Figure 5). The soil-property axis and SMF were low in early stages and higher from the tree–shrub stage onward, whereas the leaf economic and functional diversity axes followed more variable trajectories (Figure 5A). The recovery stage was strongly correlated with the soil-property axis (r = 0.87, *p* < 0.001) and SMF (r = 0.88, *p* < 0.001), and was also associated with the leaf economic axis (r = 0.61, *p* = 0.007) and the display-oriented functional-diversity axis (r = 0.68, *p* < 0.01) (Figure 5B). SMF was strongly correlated with the soil-property axis (r = 1.00, *p* < 0.001) and the display-oriented functional-diversity axis (r = 0.66, *p* < 0.01), whereas its relationship with the leaf economic axis was weaker (r = 0.43). The corresponding coefficients for the original functional-diversity PC1 have negative signs because that axis was multiplied by −1 only for Figure 5. Because the soil-property axis and SMF were derived from the same indicators, their strong association primarily reflects internal consistency. Overall, variation in the SMF was most closely aligned with the measured soil-property gradient, while plant functional reorganization provided complementary evidence of differences between early resource-poor and later higher-scoring stages (Figure 5C).

## 3. Discussion

### 3.1. Stage-Related SMF Patterns After Woody-Structure Establishment

Across the sampled recovery sequence, soil bulk density was lower, and soil moisture, nutrient contents, and the SMF were higher from the tree–shrub stage onward (Figure 1A,B). This pattern is consistent with the interpretation that differences in vegetation physiognomy and community structure were accompanied by differences in soil physical properties, moisture status, and nutrient contents. In the soil-property PCA, stage centroids shifted from grassland and grass–shrub plots toward tree and near-climax plots, which had higher integrated soil-property scores (Figure 1C). Ecological engineering in southwestern China’s karst landscapes has increased vegetation cover and carbon stocks, although greening does not necessarily coincide with simultaneous soil functional recovery [9]. Recent studies also show that recovery age, carbon sequestration, and water retention are associated with soil-function changes in karst systems [27,28]. Our results extend this evidence by showing that higher SMF values were concentrated in the tree–shrub, tree, and near-climax stages. This represents a pronounced spatial stage-related shift associated with woody-plant establishment, not a statistically identified temporal threshold from structural to functional recovery.

The stage-related pattern may reflect the strong resource limitation of karst habitats. Karst landscapes have shallow soils, extensive bedrock exposure, and pronounced fissure drainage, and early herbaceous or low-shrub communities may not yet provide stable litter inputs, root networks, or canopy buffering [8]. Accordingly, the low SMF values in HE and HS were associated with lower soil moisture, organic carbon, and nutrient contents (Figure 1A,B). Studies in karst regions likewise show that relationships between vegetation recovery, soil carbon pools, microbial networks, and soil multifunctionality vary among successional stages [10,29]. Similar patterns have been observed in other karst regions. In Mediterranean karst landscapes, organic amendments in limestone quarry restoration increased soil organic carbon and plant nutrients while improving soil aggregation [30]. In tropical karst ecosystems of the Brazilian savannah, pasture restoration has been shown to reduce runoff, soil loss, and sediment enrichment, further supporting the spatial association between vegetation recovery and improved soil and hydrological conditions in karst landscapes [31]. These convergent patterns suggest that the association between vegetation recovery and soil property improvement may be a general feature of karst ecosystem restoration across different climatic and geographic contexts. Large-scale restoration can also alter trade-offs and synergies among ecosystem services [32]. By constructing the SMF only from soil physical, moisture, and carbon and nutrient content indicators, this study avoids conflating the proxy with broad ecosystem-level multifunctionality and permits a more specific interpretation of soil property patterns.

### 3.2. Plant Functional Reorganization Is Associated with Environmental Filtering and Strategy Reshuffling

The leaf economic spectrum results show that natural recovery of karst forests was accompanied by stage-related reorganization of dominant plant functional strategies. Recovery stages were differentiated in community-weighted leaf-trait space, and their centroids shifted along the recovery sequence (Figure 2A). SLA, LNC, and LPC contributed in the opposite direction to LDMC, LA, and LCC on the primary leaf economic axis, which may reflect trade-offs among resource acquisition, nutrient use, and tissue investment (Figure 2C). Consistent with leaf economic spectrum theory, higher SLA and leaf nutrient contents generally indicate rapid resource acquisition, whereas higher LDMC and greater tissue investment correspond to resource conservation [17,18]. Intraspecific leaf-trait variation, neighborhood heterogeneity, and local light conditions can also influence tree growth and community functional performance [33,34]. The significant stage-related variation in the integrated leaf economic axis therefore suggests not only species turnover but also reconfiguration of dominant functional strategies (Figure 2B–D).

The integrated leaf economic axis was not monotonic across the recovery sequence but fluctuated during transitional stages such as grass–shrub and tree–shrub (Figure 2B). This differs from the simple acquisitive-to-conservative shift reported in some forest succession studies and may partly reflect the pronounced spatial heterogeneity of karst habitats. Shallow soils, fissure water, patchy nutrients, and variable bedrock exposure can filter both acquisitive and stress-tolerant species, allowing multiple functional strategies to coexist during intermediate stages. Plant trait combinations in southwestern karst forests are jointly related to soil resources, topographic heterogeneity, and environmental filtering [35,36], while topographic complexity and hydraulic-trait variation can modify understory trait composition and tree stress resistance [37,38]. The observed leaf-economic-spectrum pattern should therefore be interpreted as stage-related strategy reshuffling rather than a simple transition from fast to slow strategies.

Functional diversity results further showed that not all dimensions of functional space changed synchronously across the recovery sequence. Recovery stages were differentiated in functional-diversity space, and the integrated axis varied among stages (Figure 3A,B). FRic, FDis, and RaoQ showed more pronounced stage variation, whereas FEve and FDiv followed more complex patterns (Figure 3C,D). These metrics represent different facets of functional structure, including functional-space range, evenness, divergence, and abundance-weighted dissimilarity [23,24]. Their contrasting responses explain why the association between functional diversity and the SMF cannot be summarized as “higher functional diversity equals stronger soil function.” Expansion of functional space during early and intermediate recovery may broaden resource use, whereas woody dominance and stronger environmental filtering in later stages may produce convergence in some dimensions. Assembly history and restoration stage can alter biodiversity–function relationships [39,40], and functional composition across forest succession may reflect both niche complementarity and mass-ratio effects [41]. Karst grassland studies likewise show that functional-diversity effects depend on recovery stage and context [42]. The simultaneous occurrence of functional-space expansion and partial convergence in our study therefore suggests that higher SMF values were more closely associated with stable dominant trait combinations than with a continuous increase in functional dissimilarity.

### 3.3. Functional-Module Associations and Restoration Implications

A central finding is that variation in the SMF was not attributable to a single leaf trait or functional-diversity index. Instead, it was associated with the measured soil properties, leaf economic spectrum, and functional-diversity modules. SLA and FRic had strong positive correlations with the SMF, whereas several dispersion-related indices had weak or negative associations (Figure 4A). Plant functional indicators also differed in the direction and magnitude of their associations with soil moisture, organic carbon, nutrient contents, and SMF (Figure 4B). Comparable patterns occur in multifunctionality research, in which plant functional composition and diversity may be associated with functioning through resource-use complementarity, litter inputs, and nutrient cycling [43,44]. Forest experiments further show that structural complexity, understory shrub diversity, and multitrophic diversity can modify productivity and multifunctionality [45,46] and that functional traits regulate tree growth and biotic interactions [47]. Greater functional dissimilarity therefore does not necessarily correspond to a higher SMF, underscoring the indicator-dependent nature of these associations. This soil-centered, transparent, and sample-dependent composite index serves as an intermediate approach between single-indicator methods and excessively broad multifunctionality assertions. Its value lies in its operational simplicity and straightforward interpretability for assessing karst restoration.

Differences among indicators may arise from stronger environmental filtering and more stable dominant functional assemblages during later recovery stages. We speculate that, in stages with higher SMF values, relatively stable woody structure may be associated with continuous root inputs, litter accumulation, canopy buffering, and microenvironmental stability, although these processes were not directly measured in this study. The r = 1.00 association between the soil-property axis and SMF is mathematically non-independent because both were calculated from the same six variables (Figure 5B). Biodiversity–multifunctionality relationships are strongly context-dependent and can be influenced by community age, nutrient availability, and dominant functional composition [7,48]. Tree traits, seed-family performance, and functional redundancy can further affect community performance and functional stability [49,50]. The positive FRic–SMF association may therefore reflect a contribution of functional-space range to resource use, whereas weak or negative associations involving FDis and RaoQ may reflect strategy differentiation at high dispersion. Collectively, the results indicate that the SMF was associated with the measured soil-property gradient, reorganization of the leaf economic spectrum, and restructuring of functional space; concordance between the soil axis and SMF is an internal feature of index construction rather than independent evidence of ecological coupling (Figure 5A–C).

In contrast to many studies of karst restoration, this study operationally defined an SMF from soil physical structure, moisture status, and carbon and nutrient contents and integrated it with the leaf economic spectrum and functional diversity. Previous work has mainly examined individual soil properties, carbon storage, or microbial communities, and relatively few studies have considered plant functional strategies, functional-space structure, and soil multifunctionality together [2,51]. Karst forest studies also show that diversity, soil chemistry, and bedrock exposure influence forest structure and multifunctionality [51,52], while evidence from other degraded ecosystems indicates that agroforestry, plantations, and community functional composition can affect ecosystem services and multifunctionality [53,54]. Restoration assessments should therefore not rely only on vegetation cover, species richness, or isolated soil indicators, but should evaluate whether plant functional reorganization is associated with improved soil structure, moisture status, and nutrient contents (Figure 1, Figure 2, Figure 3, Figure 4 and Figure 5). Higher SMF values from the tree–shrub stage onward identify stages associated with improved soil-property conditions but do not establish a temporal threshold (Figure 1B and Figure 5A). Rocky desertification control should also consider trade-offs, synergies, and optimization among ecosystem services [55]. Tree diversity may enhance canopy density, structural complexity, and carbon stocks [56,57]; management could therefore prioritize conditions that support woody regeneration, litter accumulation, root inputs, and stable functional assemblages. A sensitivity analysis using PCA-derived weights further confirmed that the choice of weighting scheme did not alter the main conclusions. The principal limitations are the small sample size (18 plots), the space-for-time design, and the use of static soil-property proxies rather than direct process rates. Long-term monitoring, root traits, soil microbial processes, and litter-decomposition measurements are needed to test the mechanisms proposed here [58,59]. Furthermore, comprehensive abiotic covariates—such as aspect, topographic position, wind exposure, and high-resolution climatic data—were unavailable in the current dataset, as the study was not initially designed to include these measurements. Future research incorporating these variables, potentially summarized through principal component analysis or factor analysis, would facilitate a more rigorous distinction between recovery effects and environmental heterogeneity. Importantly, since our study utilized a space-for-time substitution design involving 18 plots, all observed relationships should be interpreted as spatial associations along the operational recovery gradient, rather than as direct evidence of temporal succession, causal effects, or statistically defined restoration thresholds.

## 4. Materials and Methods

### 4.1. Study Area

**Figure 6:**
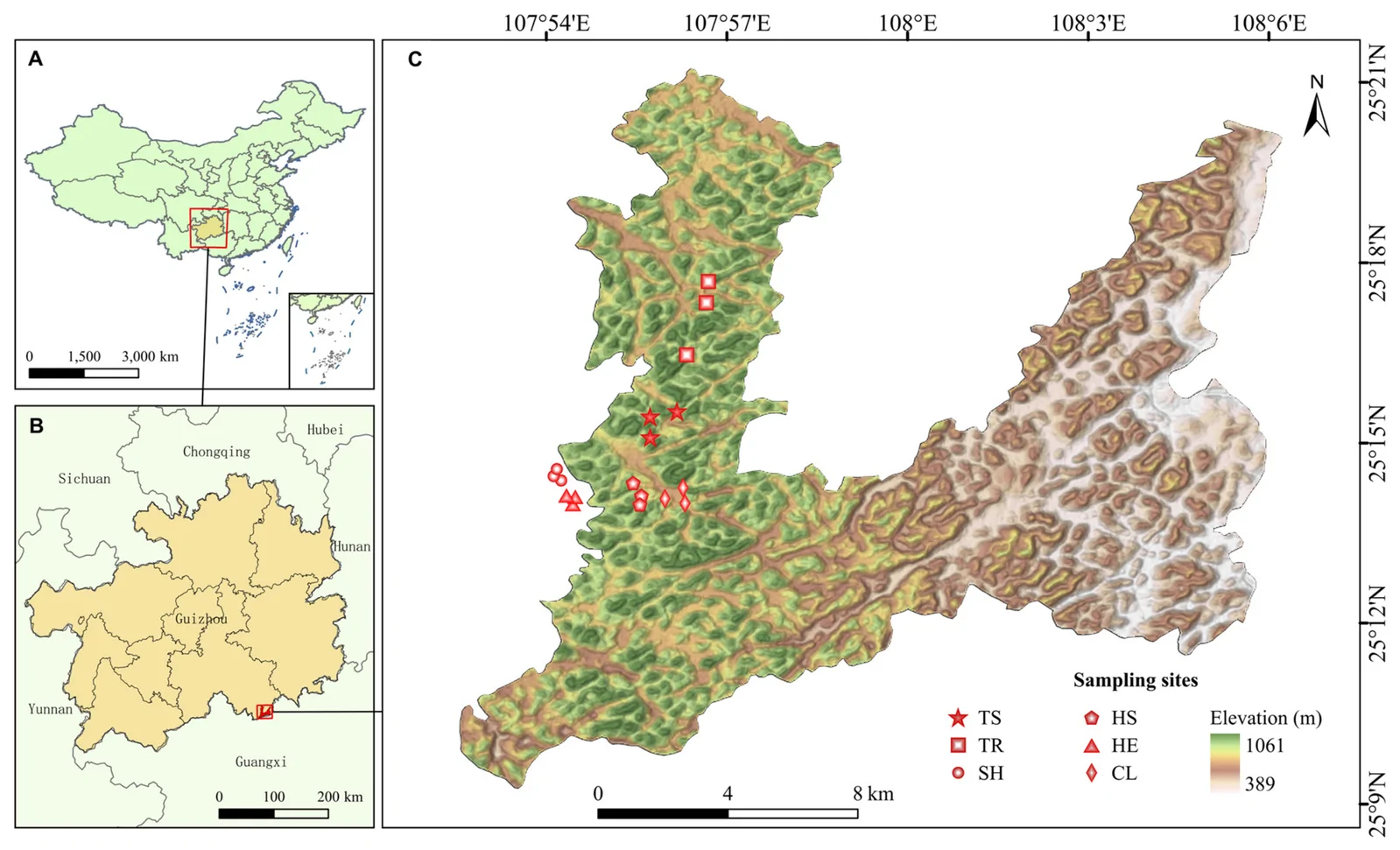
Location of the Maolan karst forest study area and sampling plots. (**A**) Map of China’s administrative divisions, (**B**) Map of Guizhou Province’s administrative divisions, (**C**) Maolan Karst National Nature Reserve. Plots represent six recovery stages: grassland (HE), grass–shrub (HS), shrubland (SH), tree–shrub (TS), tree (TR), and near-climax community (CL). Three independent plots were established per stage.

The study was conducted in the Maolan karst forest region of Libo County, Qiannan Buyi and Miao Autonomous Prefecture, Guizhou Province, southwestern China. The region lies within a core karst zone at approximately 107°52′10″–108°05′40″ E and 25°09′20″–25°20′50″ N, with elevations of approximately 430–1078.6 m. The climate is a humid subtropical monsoon climate, with warm and wet growing seasons that support well-preserved forest vegetation. The landscape is dominated by karst peak clusters, depressions, and erosional hills. Carbonate rocks are extensively exposed, and the predominantly calcareous soils are shallow and discontinuous, with limited water- and nutrient-retention capacity. The Maolan region contains a relatively complete natural karst forest recovery sequence and is therefore suitable for examining associations between plant functional structure and soil-property patterns during vegetation recovery. Sampling locations are shown in Figure 6.

### 4.2. Plot Establishment and Classification of Recovery Stages

A space-for-time substitution approach was employed to develop an operational natural recovery sequence within the Maolan karst forest region. Plots were categorized into six recovery stages—grassland (HE), grass–shrub (HS), shrubland (SH), tree–shrub (TS), tree (TR), and near-climax community (CL)—based on vegetation physiognomy, dominant life forms, woody plant development, community cover, dominant species composition, and community recovery status. Three plots were established for each stage, resulting in a total of 18 plots.

The six-stage classification was developed not solely based on site location or topographic characteristics but was grounded in established successional frameworks previously applied to the Maolan karst forests. Yu et al. [60] identified a comparable six-stage natural recovery sequence in this region, and Huang et al. [61] employed a similar framework to investigate carbon sequestration along the Maolan natural restoration gradient. Consequently, the stage classification was founded on recognized regional vegetation succession criteria rather than on arbitrary site groupings.

To minimize confounding effects arising from abiotic site factors, plots were selected within a single nature reserve characterized by a consistent carbonate lithological background and broadly comparable disturbance histories. For each plot, longitude, latitude, elevation, slope, bedrock exposure, community cover, and dominant species were documented (see Table 1 and Table 2). Nonetheless, karst landscapes are intrinsically heterogeneous, and measurable variations in elevation, slope, bedrock exposure, soil depth, and microtopography may still affect vegetation and soil properties. Consequently, the stage-related differences observed in this study are interpreted as spatial associations along an operational recovery gradient rather than as definitive evidence of long-term temporal succession, causal relationships, or statistically established restoration thresholds.

### 4.3. Vegetation Survey and Community-Weighted Leaf Traits

Vegetation surveys recorded species identity, cover, individual count, plant height, crown width, and dominance within each plot (Table 2). For trees and shrubs, abundance, height, crown width, and diameter at breast height or basal diameter were measured. For herbaceous plants, species composition, cover, and mean height were recorded. Measurements were adapted to life form and community structure, whereas the 30 × 30 m plot framework and within-stage protocols remained consistent. A composite species weight was calculated from the available relative density, relative cover, and relative height components and was normalized within each plot so that species weights summed to one. For herbaceous plots in which some components were unavailable, the consistently recorded relative-cover or relative-dominance measure was normalized within the plot and applied consistently across all herbaceous plots.

Community-weighted mean (CWM) traits were used to represent dominant functional strategies at the plot scale and were calculated as follows:

(1) $${CWM}_{tj}=\sum p_{ij}x_{it}$$

where *CWM_tj_* is the community-weighted mean of trait *t* in plot *j*, *p_ij_* is the normalized weight of species *i* in plot *j*, and *x_it_* is the value of trait *t* for species *i*. CWM values emphasize dominant species and provide plot-level measures of community functional strategies [16].

### 4.4. Measurement of Leaf Functional Traits

Leaf functional traits were measured following standardized protocols [62,63]. During the peak growing season, healthy mature individuals of dominant and common associated species were selected within each plot. For each species, three to five healthy plants without visible pest or disease damage were sampled. Fully expanded mature leaves were collected from the upper-middle canopy under comparable light conditions, placed immediately in sealed bags, transported to the laboratory in a cooled container, and measured as soon as possible to minimize water loss.

Leaf area (LA) was determined by scanning leaves with a flatbed scanner (Epson Perfection V600 Photo, Epson, Nagano, Japan) and analyzing the images in ImageJ (National Institutes of Health, Bethesda, MD, USA, v1.54). Leaf thickness (LT) was measured at two or three locations per leaf, excluding the main vein, with a digital caliper (Mitutoyo, Kawasaki, Japan), and the mean was used. Saturated fresh mass was measured with an electronic balance (Sartorius, Göttingen, Germany). Leaves were then dried at 75 °C to constant mass in a forced-air drying oven (DHG series, Shanghai Yiheng Scientific Instrument Co., Ltd., Shanghai, China), and dry mass was recorded. Specific leaf area (SLA) was calculated as leaf area divided by leaf dry mass, and leaf dry matter content (LDMC) as leaf dry mass divided by saturated fresh mass. Leaf carbon content (LCC) and leaf nitrogen content (LNC) were measured with an elemental analyser (Vario EL cube, Elementar, Langenselbold, Germany). Leaf phosphorus content (LPC) was determined using the molybdenum–antimony colorimetric method after acid digestion and a UV–visible spectrophotometer (UV-2600, Shimadzu, Kyoto, Japan).

### 4.5. Soil Sampling and Measurement of Soil-Property Indicators

In each plot, surface soils were sampled at 0–20 cm depth using a five-point composite method. Surface litter, stones, and coarse roots were removed before sampling. Soil from five representative microsites was thoroughly mixed to form one composite sample per plot. Each composite sample was divided into two portions. One portion of fresh soil was sealed and stored at low temperature for determination of gravimetric moisture content. The other portion was air-dried, cleared of visible roots and stones, and passed through a 2 mm sieve for physicochemical analyses. A subsample was further ground and passed through a 0.149 mm sieve for carbon, nitrogen, phosphorus, and potassium analyses.

Soil bulk density (BD) was determined from 100-cm^3^ undisturbed cores using the cutting-ring method; cores were oven-dried at 105 °C to constant mass. Soil gravimetric moisture content (SPMC) at sampling was determined by oven drying and represents instantaneous soil moisture status rather than water-holding capacity. Soil organic carbon (SOC) was measured by dichromate oxidation with external heating. Soil nitrogen content (SNC) was determined by the Kjeldahl method, soil phosphorus content (SPC) by the molybdenum–antimony colorimetric method after acid digestion, and soil potassium content (SKC) by flame photometry after acid digestion. Analyses followed standard soil chemical procedures [64]. SOC, SNC, SPC, and SKC are measured contents and were not converted to area-based stocks.

### 4.6. Calculation of Functional Diversity

Functional diversity was quantified from the species-by-trait matrix and plot-by-species normalized weight matrix after trait Z-score standardization. Five indices were selected to characterize community functional space: functional richness (FRic), functional evenness (FEve), functional divergence (FDiv), functional dispersion (FDis), and Rao’s quadratic entropy (RaoQ). FRic is the volume of functional space occupied by a community. FEve describes the evenness of species and abundance distribution within that space. FDiv measures the extent to which dominant weights occur toward the periphery of functional space. FDis is the abundance-weighted mean distance of species from the community centroid, and RaoQ is the abundance-weighted functional dissimilarity among species [23,24].

(2) $${FE}_{ve}=\frac{\sum_{i=1}^{S-1}\min\left({PEW}_{i},\frac{1}{S-1}\right)-\frac{1}{S-1}}{1-\frac{1}{S-1}}$$

where *S* is species richness and *PEW_i_* is the partial weighted evenness of branch i in the minimum spanning tree of functional trait space.

(3) $${FD}_{is}=\sum \frac{\sum_{i=1}^{S}a_{ij}z_{ij}}{\sum_{i=1}^{S}a_{ij}}$$

where *a_ij_* is the normalized weight of species *i* in plot j, and *z_ij_* is the distance from species *i* to the abundance-weighted centroid of plot j in standardized multidimensional trait space.

(4) $${FD}_{iv}=\frac{2}{\pi }arctan\left\{5\times \sum_{i=1}^{N}\left[{\left(\ln C_{i}-\ln\bar{x}\right)}^{2}\right]\times A_{i}\right\}$$

where *C_i_* is the distance of species i from the centroid of functional trait space; $\bar{x}$ is the abundance-weighted mean distance of all species from that centroid; and *A_i_* is the normalized weight of species *i*.

(5) $${FR}_{ic}=\frac{{SF}_{ic}}{R_{c}}$$

where *SFic* is the functional space occupied by the community, and *Rc* is the total functional space defined by the trait matrix.

(6) $${RaoQ}_{j}=\sum_{i=1}^{S}\sum_{k=1}^{S}p_{ij}p_{kj}p_{ik}$$

where *RaoQ_j_* is Rao’s quadratic entropy for plot *j*; *p_ij_* and *p_kj_* are the normalized weights of species i and k in plot j; and *d_ik_* is their pairwise functional distance. All indices were calculated from Z-standardized trait values and plot-level species weights using the dbFD function in the R package FD (v1.0.12, R Foundation for Statistical Computing, Vienna, Austria).

### 4.7. Construction of the Soil Multifunctionality Proxy

Because the measured variables described soil physical structure, moisture status, and carbon and nutrient contents, we used a relative soil multifunctionality proxy (SMF) rather than claiming broad ecosystem multifunctionality. The SMF was derived from six soil-property indicators: soil bulk density (BD), soil gravimetric moisture content (SPMC), soil organic carbon (SOC), soil nitrogen content (SNC), soil phosphorus content (SPC), and soil potassium content (SKC). SPMC, SOC, SNC, SPC, and SKC were treated as positive indicators, with higher values representing greater moisture status or higher carbon and nutrient contents. BD was treated as a negative indicator because higher bulk density generally indicates lower porosity and poorer soil structure. The resulting SMF is a relative, sample-dependent composite of the measured properties; it does not directly quantify water-holding capacity, area-based nutrient or carbon stocks, carbon sequestration rates, or unmeasured ecosystem processes.

Positive indicators were min–max normalized to a 0–1 scale, and BD was reverse-normalized. The SMF was calculated as the mean of the six normalized indicators:

(7) $${SMF}_{i}=\frac{\left[{BD}_{i}^{-}+{SPMC}_{i}^{+}+{SOC}_{i}^{+}+{SNC}_{i}^{+}+{SPC}_{i}^{+}+{SKC}_{i}^{+}\right]}{6}$$

where *SMF_i_* is the soil multifunctionality proxy for plot *i*; *SPMC*^+^, *SOC*^+^, *SNC*^+^, *SPC*^+^, and *SKC*^+^ are min–max normalized soil moisture and carbon or nutrient-content indicators; and BD^−^ is reverse-normalized soil bulk density. Positive indicators were min–max normalized as *X*^+^ = (*X* − *Xmin*)/(*Xmax* − *Xmin*), and BD as *BD^−^* = (*BDmax* − *BD*)/(*BDmax* − *BDmin*). This transparent averaging approach is widely used in multifunctionality research and represents the integrated level of multiple proxies within the sampled recovery sequence, particularly when the sample size is limited [65]. To assess the robustness of the equal-weighting scheme, we conducted a sensitivity analysis using PCA-derived weights (based on the loadings of the first principal component) to recalculate the SMF. The nearly perfect correlation between the two weighting approaches supported the use of the simpler equal-weighting method (see Section 2). Equal weighting was retained as the primary approach because it avoids overfitting to the specific dataset, enhances comparability with previous multifunctionality studies, and does not require assumptions about the latent structure of the measured soil variables.

### 4.8. Statistical Analysis

All statistical analyses were conducted using R, with plots serving as the statistical units. The recovery stage was considered an ordered categorical variable, following the sequence HE, HS, SH, TS, TR, and CL. Prior to analysis, soil properties, community-weighted mean (CWM) leaf traits, functional diversity indices, and the SMF were examined for missing values, outliers, distributional characteristics, and the assumptions underlying stage-wise comparisons.

Continuous variables employed in principal component analysis (PCA) and heatmap visualization were standardized using Z-scores. The assumption of normality was assessed via the Shapiro–Wilk test, while homogeneity of variances was evaluated using Levene’s test. When both assumptions were met, differences among recovery stages were examined using one-way analysis of variance (ANOVA), followed by Tukey’s honestly significant difference (HSD) post hoc test. In cases where one or both assumptions were violated, the Kruskal–Wallis test was applied, followed by rank-based pairwise comparisons with adjustment for multiple testing using the Benjamini–Hochberg false discovery rate procedure [66]. For comparisons among recovery stages, we reported effect sizes to complement *p*-values. For one-way ANOVA, partial η^2^ was calculated. Effect sizes were interpreted following Cohen’s [67] benchmarks (small: ≥0.01; medium: ≥0.06; large: ≥0.14).

Principal Component Analysis (PCA) was performed independently on the soil-property, community-weighted mean (CWM) leaf trait, and functional-diversity matrices to derive integrated axes that represent variation in soil properties, the leaf economic spectrum, and functional-diversity structure, respectively. Spearman rank correlation analyses were employed to evaluate the relationships among recovery stage, leaf traits, functional diversity indices, the soil multifunctionality proxy (SMF), and individual soil properties. For key associations, correlation coefficients along with 95% confidence intervals were reported. Additionally, the false discovery rate was controlled using the Benjamini–Hochberg procedure [66]. For each pair of modules depicted in Figure 4C, the proportion of significant associations was determined by calculating the percentage of correlations that remained statistically significant following the Benjamini–Hochberg false discovery rate correction. Given the variability in module sizes and the limited sample size, these percentages were interpreted as descriptive summaries rather than as directly comparable effect sizes.

The three principal component analysis (PCA) axes were subsequently employed as integrated representations of the soil-property, leaf-economic-spectrum, and functional-diversity modules. For the purpose of the integrated display in Figure 5 only, the sign of the functional-diversity PC1 axis was reversed so that higher displayed scores corresponded to the direction of later recovery stages; this transformation affected only the sign, without altering the magnitude or the *p* value of its correlations. Given that the soil-property PCA axis and the SMF were derived from the same six soil variables, their correlation is mathematically non-independent and was therefore interpreted as an internal consistency check rather than as independent ecological evidence. Figure 5C serves as a descriptive synthesis of reported associations and does not constitute an additional inferential test or causal model. Statistical significance was defined as *p* < 0.05.

## 5. Conclusions

This study demonstrates that variation in soil properties during karst forest recovery is spatially associated with coordinated changes in plant functional structure, thereby challenging the simplistic assumption that increased diversity uniformly enhances ecosystem multifunctionality. The asynchronous responses observed in functional richness and dispersion metrics indicate that recovery processes involve both niche differentiation and environmental filtering. Notably, functional space volume emerges as a more informative indicator than trait dispersion. For restoration practice, we recommend transitioning from vegetation greening approaches toward trait- and function-based monitoring frameworks that directly assess below-ground recovery dynamics. In this context, functional richness and woody regeneration should be prioritized as key indicators in the management of rocky desertification. It is important to note that these findings are correlative and limited by the space-for-time substitution design, a small sample size, and the lack of direct measurements of biological processes and below-ground traits. Future research should incorporate these aspects to elucidate mechanistic links between functional reorganization and soil multifunctionality.

## Figures and Tables

**Table 1:** Characteristics of plots along the karst forest recovery sequence.

Site	Plot Size (m × m)	Longitude (°)	Latitude (°)	Elevation (m)	Slope (°)	Bedrock Exposure (%)
HE1	30 × 30	107.907346	25.233396	822	17.3	65
HE2	30 × 30	107.908011	25.235425	825	18.8	68
HE3	30 × 30	107.905581	25.235799	845	19.2	70
HS1	30 × 30	107.924047	25.239072	836	15.2	60
HS2	30 × 30	107.926335	25.235548	849	14.3	58
HS3	30 × 30	107.925899	25.233114	802	14.2	55
SH1	30 × 30	107.904123	25.239665	865	26.1	54
SH2	30 × 30	107.902035	25.240866	858	22.3	52
SH3	30 × 30	107.902924	25.242822	863	24.6	53
TS1	30 × 30	107.928781	25.251595	763	35.6	65
TS2	30 × 30	107.928756	25.257348	778	42.3	60
TS3	30 × 30	107.936189	25.258795	802	45.4	62
TR1	30 × 30	107.944872	25.294787	710	27.3	33
TR2	30 × 30	107.938855	25.27445	717	29.9	30
TR3	30 × 30	107.944314	25.288926	963	23.6	29
CL1	30 × 30	107.937844	25.237649	669	30.2	22
CL2	30 × 30	107.938375	25.233347	693	24.5	25
CL3	30 × 30	107.932841	25.234604	729	26.3	23

**Table 2:** Community structure and dominant species across recovery stages.

Recovery Stage	Dominant Species	Community Cover (%)	Mean Height (m)	Relative Abundance (%)
Grassland (HE)	*Miscanthus sinensis*, *Cyclosorus interruptus*, *Ficus tikoua*	55	0.21 ± 0.05	64
Grass–shrub (HS)	*Nandina domestica*, *Tirpitzia sinensis*, *Selaginella uncinata*	65	1.11 ± 0.32	72
Shrubland (SH)	*Loropetalum chinense*, *Nandina domestica*, *Athyrium filix-femina*, *Carex capilliformis*	70	1.35 ± 0.12	75
Tree–shrub (TS)	*Lindera communis*, *Nandina domestica*	80	3.56 ± 0.49	81
Tree (TR)	*Cornus wilsoniana*, *Lindera communis*, *Nandina domestica*	85	8.9 ± 0.54	85
Near-climax community (CL)	*Cornus wilsoniana*, *Emmenopterys henryi*, *Castanopsis fargesii*	92	11.77 ± 0.88	90

## Data Availability

The original contributions presented in the study are included in the article, further inquiries can be directed to the corresponding author.

## Supplementary Materials

The following supporting information can be downloaded at: https://www.mdpi.com/article/10.3390/plants15162448/s1, Table S1. Mean (±standard error) of soil properties and the soil multi-functionality (SMF) proxy across six recovery stages in the Maolan karst forests.

## Abbreviations

BD, soil bulk density; CWM, community-weighted mean; FDis, functional dispersion; FDiv, functional divergence; FEve, functional evenness; FRic, functional richness; LA, leaf area; LCC, leaf carbon content; LDMC, leaf dry matter content; LNC, leaf nitrogen content; LPC, leaf phosphorus content; LT, leaf thickness; PCA, principal component analysis; RaoQ, Rao’s quadratic entropy; SKC, soil potassium content; SMF, soil multifunctionality proxy; SNC, soil nitrogen content; SOC, soil organic carbon; SPC, soil phosphorus content; SPMC, soil gravimetric moisture content; SLA, specific leaf area; HE, grassland; HS, grass–shrub; SH, shrubland; TS, tree–shrub; TR, tree; CL, near-climax community.
