# Supplementary material for: Links Between Plant Functional Reorganization and a Soil Multifunctionality Proxy During Karst Forest Recovery
Source: Plants (Basel). 2026 Aug 12;15(16):2448. doi: 10.3390/plants15162448 (PMC13516893; doi:10.3390/plants15162448)
Supplement: Supplementary file 1 [file plants-15-02448-s001.zip › plants-4482008-supplementary.pdf]

Supplementary Table S1. Mean (± standard error) of soil properties and the soil multifunctionality (SMF) proxy across six recovery stages in the Maolan karst forests

Stage	BD ($g \cdot cm^{-3}$)	SPMC (%)	SOC ($g \cdot kg^{-1}$)	SNC ($g \cdot kg^{-1}$)	SPC ($g \cdot kg^{-1}$)	SKC ($g \cdot kg^{-1}$)	SMF
HE	1.193 ± 0.049	17.9 ± 0.8	22.4 ± 2.0	0.872 ± 0.057	0.313 ± 0.045	3.87 ± 0.14	0.063 ± 0.019
HS	0.874 ± 0.050	18.6 ± 0.5	49.5 ± 7.2	1.924 ± 0.278	0.615 ± 0.026	4.44 ± 0.13	0.272 ± 0.032
SH	1.121 ± 0.023	19.5 ± 1.3	24.0 ± 2.5	0.493 ± 0.043	1.132 ± 0.189	5.91 ± 0.31	0.214 ± 0.037
TS	0.726 ± 0.036	29.9 ± 2.2	82.9 ± 0.3	3.860 ± 0.188	0.995 ± 0.198	5.61 ± 0.07	0.596 ± 0.017
TR	0.759 ± 0.080	30.1 ± 4.8	60.3 ± 3.5	2.256 ± 0.263	1.514 ± 0.104	7.28 ± 0.12	0.609 ± 0.041
CL	0.615 ± 0.028	40.8 ± 3.4	76.1 ± 3.5	3.690 ± 0.209	2.005 ± 0.154	4.68 ± 0.08	0.737 ± 0.034
